# Immunohistochemistry for Myc Predicts Survival in Colorectal Cancer

**DOI:** 10.1371/journal.pone.0087456

**Published:** 2014-02-04

**Authors:** Christopher W. Toon, Angela Chou, Adele Clarkson, Keshani DeSilva, Michelle Houang, Joseph C. Y. Chan, Loretta L. Sioson, Lucy Jankova, Anthony J. Gill

**Affiliations:** 1 Cancer Diagnosis and Pathology Group, Kolling Institute of Medical Research, Sydney, Australia; 2 Histopath Pathology, North Ryde, Australia; 3 Dept of Anatomical Pathology, SYDPATH, St Vincents Hospital, Darlinghurst, Australia; 4 Dept of Anatomical Pathology, Royal North Shore Hospital, St Leonards, Australia; 5 University of Sydney, Camperdown, Australia; 6 Bill Walsh Cancer Research Laboratory, Kolling Institute of Medical Research, Sydney, Australia; University of Illinois at Chicago, United States of America

## Abstract

MYC over-expression as determined by molecular means has been reported as a favorable prognostic biomarker in colorectal carcinoma (CRC). However MYC expression analysis is not available in the routine clinical setting. We investigated whether immunohistochemistry (IHC) for the myc protein using a novel commercially available rabbit monoclonal antibody [clone Y69] which is currently in widespread clinical use for lymphoma diagnosis could be used to predict outcome in resected CRC. Myc IHC was performed on a tissue microarray (TMA) comprising a retrospective cohort of 1421 CRC patients and scored blinded as to all clinical and pathological data. IHC was also performed on a subcohort of whole section CRCs to assess staining characteristics and concordance with TMA expression. MYC over-expression was found in 980 (69%) of CRCs and was associated with tumor stage and DNA mismatch repair/BRAF status. There was substantial agreement between TMA and whole section myc IHC (kappa = 0.742, p<0.01). CRCs with MYC over-expression demonstrated improved 5-year survival (93.2% vs. 57.3%), with the effect significantly modulated by the dominant effect of tumor stage, age at diagnosis and lymphovascular space invasion status on survival. We conclude that myc status as determined by IHC alone can be used to predict overall survival in patients with CRC undergoing surgical resection.

## Introduction

Dysregulation of MYC (HGNC:7553) is an early consequence of activating mutations in APC(HGNC:583), a key driver mutation in the adenoma-carcinoma pathway in colorectal cancer (CRC). Mediated by beta-catenin via the canonical Wnt pathway, aberrant over-expression of MYC and CYCLIN D1 results in uncontrolled cellular proliferation, conferring a growth advantage to cancer cells [1], [2]. The role of MYC over-expression in driving CRC tumorigenesis has been confirmed by gene expression profiling studies [3], and more recently by CRC genome-wide analysis as part of the Cancer Genome Atlas Network initiative [4].

Interestingly MYC over-expression as determined by Northern blot analysis was reported as a biomarker of good outcome in colorectal cancer as far back as 1996. [5] Currently its use as a prognostic biomarker is difficult to justify because of the limited availability and expense involved of molecular testing. Therefore testing for MYC over-expression in CRC is not currently available, feasible or warranted in the routine clinical setting.

Recently a novel highly sensitive and specific rabbit monoclonal antibody directed against the myc protein (clone Y69) has become available. This antibody has been validated in the routine clinical setting in formalin fixed paraffin embedded tissue, where myc immunohistochemsitry in combination with bcl2 immunohistochemistry, has been used to identify the poor prognostic subgroup of non-Hodgkin lymphoma known as ‘double hit lymphoma’. [6], [7] In fact MYC and BCL2 expression as determined by immunohistochemsitry in formalin fixed paraffin embedded tissue has rapidly become part of the routine diagnostic assessment of patients with high grade B-cell lymphoma. [8].

We sought to revisit the use of MYC expression as biomarker in CRC by correlating outcome with MYC expression as determined by this widely available rabbit monoclonal antibody.

## Materials and Methods

Our CRC cohort has been previously described. [9] Briefly, retrospective CRC cases were recruited by searching the pathology database of the Department of Anatomical Pathology, Royal North Shore Hospital, Sydney for patients who had definitive operations for CRC between 2004 and 2009. During this period this center performed centralized pathological testing for two quaternary referral hospitals with dedicated colorectal surgery units and 4 community hospitals. Therefore this patient cohort represents a true snapshot of CRC cases encountered in the Australian community as a whole.

Exclusion criteria were tumors of extra-colonic and appendiceal location, tumors treated exclusively endoluminally and histological types other than adenocarcinoma defined by the WHO 2010 system. [10].

Tumors were independently reviewed by three pathologists (CT, KDS and AG) to confirm the diagnosis and to restage the tumors according to 7^th^ edition 2009 AJCC/TNM. [11] For resections involving synchronous tumors, the tumor with the highest pathologic stage was selected and annotated. Tissue microarrays (TMA) containing duplicate 1 mm cores were then constructed from available tumor tissue blocks.

Immunohistochemistry was performed on the TMA using a commercially available rabbit anti-myc antibody (clone Y69, cat:ab32072, Abcam, Burlingame CA USA) using the Leica BondIII autostainer (Leica microsystems, Mount Waverley, Victoria, Australia) according to the manufacturer’s protocol. The slides were dewaxed in Bond Dewax solution (AR9222, Leica Microsystems) and hydrated in Bond Wash solution (AR9590, Leica Microsystems). Heat induced epitope retrieval was performed for 60 minutes in the manufacturer’s alkaline retrieval solution ER2 (VBS part no: AR9640, Leica Microsystems). Slides were then incubated with the primary antibody at a dilution of 1 in 100 for 30 minutes at room temperature. Antibody detection was performed using the biotin free Bond Polymer Defined Detection System (DS9713 Leica Microsystems) according to the manufacturer’s protocol. Slides were then counterstained with hematoxylin.

MYC over-expression was assessed by myc IHC, scored using a two-tiered visual system by a practicing surgical pathologist (CT) blinded to all clinical, pathological and outcome information. Nuclear staining of any intensity in greater than 10% of neoplastic cells was scored as positive (over-expressed). All other patterns of staining were scored as negative. An external control (a MYC amplified lymphoma) was run with each batch of slides.

DNA mismatch repair (MMR) and BRAF IHC status were determined by immunohistochemistry as previously described [9], [12].

In order to examine the accuracy of TMA IHC interpretation, Myc IHC was performed on whole sections of CRCs on a subcohort of 98 consecutive CRCs from 2004, interpreted by same assessor on TMA (CT) blinded to all clinical, pathological and TMA data.

Correlation between MYC over-expression and CRC patient clinicopathological variables were examined in 2×2 contingengy tables for categorical variables or using the Mann-Whitney test for age (Table 1). Variables which showed a significance of p<0.10 were included in a binary logistic regression model to further examine the relationship between MYC over-expression and DNA mismatch repair (MMR)/BRAF status IHC phenotype (Table 2).

**Table 1:** Clinical and pathological characteristics of 1421 consecutive CRC patients (2004–2009).

Variable	myc IHC positive	myc IHC negative	p-value*
**Gender, n (valid %)**			0.717
Female	514 (52.4)	226 (51.2)	
Male	466 (47.6)	215 (48.8)	
**Age at diagnosis,** median (range)	73 (17–100)	75 (33–98)	0.071
**Anatomic location, n (valid %)**			0.771
Rectum	247 (25.5)	113 (25.7)	
Caecum	218 (22.5)	94 (21.4)	
Ascending colon	142 (14.6)	76 (17.3)	
Transverse colon	119 (12.3)	48 (10.9)	
Descending colon	33 (3.4)	18 (4.1)	
Sigmoid colon	211 (21.8)	91 (20.7)	
**Histologic grade, n (valid %)**			0.755
Low	553 (79.0)	269 (80.1)	
High	147 (21.0)	67 (19.9)	
**Lymphovascular space invasion, n (valid %)**			0.663
Absent	362 (36.9)	173 (39.2)	
Present	303 (30.9)	155 (35.1)	
**Peritumoral lymphocyte reaction, n (valid %)**			0.316
Absent	34 (4.8)	11 (3.3)	
Present	669 (95.2)	326 (96.7)	
**Overall Stage AJCC/TNM 7^th^ ed, n (valid %)**			0.003
I	181 (18.5)	53 (12.0)	
IIA	298 (30.4)	116 (26.3)	
IIB	52 (5.3)	33 (7.5)	
IIC	11 (1.1)	4 (0.9)	
IIIA	49 (5.0)	15 (3.4)	
IIIB	237 (24.2)	136 (30.8)	
IIIC	116 (11.8)	58 (13.2)	
IVA	20 (2.0)	12 (2.7)	
IVB	16 (1.6)	14 (3.2)	
**MMR IHC status, n (valid %)**			0.008
Proficient	770 (78.6)	374 (84.8)	
Deficient	210 (21.4)	67 (15.2)	
**BRAFV600E IHC status, n (valid %)**			0.035
Wild type	776 (79.2)	371 (84.1)	
Mutant	204 (20.8)	70 (15.9)	
**IHC phenotypes, n (valid %)**			0.001
MMRp/BRAFwt	714 (72.9)	339 (76.9)	
MMRd/BRAFwt	61 (6.2)	32 (7.3)	
MMRd/BRAFV600E	149 (15.2)	35 (7.9)	
MMRp/BRAFV600E	56 (5.7)	35 (7.9)	

*Reports on the significance of differences between myc positive and negative groups for each variable, using either Pearson chi-square test (with continuity correction for 2×2 tables) for categorical variables or Mann-Whitney U test for age.

**Table 2:** Multivariable binary logistic regression showing adjusted effect of MMR/BRAF IHC phenotype on myc over-expression in 1421 CRCs.

Variable	Multivariable analysis
	*OR (95%CI), p*
**Age at diagnosis**	0.99 (0.98–1.00), 0.02
**Overall Stage AJCC/TNM 7^th^ edition**	
I	1.00
IIA	0.72 (0.49–1.05), 0.09
IIB	0.48 (.28–.82), <0.01
IIC	0.70 (0.21–2.34), 0.57
IIIA	0.93 (0.48–1.80), 0.83
IIIB	0.51 (0.45–0.74), <0.01
IIIC	0.60 (0.38–0.94), 0.02
IVA	0.49 (0.22–1.07), 0.08
IVB	0.34 (0.15–0.75), <0.01
**MMR/BRAF IHC phenotype**	
MMRp/BRAFwt	1.00
MMRd/BRAFwt	0.88 (0.58–1.38), 0.57
MMRd/BRAFV600E	2.17 (1.45–3.24), <0.01
MMRp/BRAFV600E	0.85 (0.54–1.34), 0.48

Follow-up was obtained by the examination of hospital medical records, those from surgeons’ private rooms and archival public death notices and obituaries in the state of New South Wales, Australia. Overall survival was defined as the duration alive from time of definitive surgery. Patients were followed up until death or their last date of follow-up not more than 7 years after definitive surgery.

Kaplan Meier analysis was used to report 5-year overall survivals for CRCs with and without MYC over-expression, with the difference assessed by Log Rank Test. Multivariable cox regression was used to explore the association between adjusted effect of MYC over-expression on overall survival, in a full model which included age at diagnosis, gender, anatomic site of tumor, tumor stage, combined mismatch repair deficiency and BRAF IHC status, tumor size, histologic grade, presence or absence of lymphovascular space invasion and peritumoral lymphocyte response status.

A p<0.05 was taken as significant, except in the initial modeling using binary logistic regression (mentioned above). Statistical analyses were performed using IBM SPSS Statistics v21 on OSX.

## Results

A total of 1421 CRCs were assessed on TMA. The clinical and pathological features are summarized in Table 1. Briefly, there was approximately equal gender distribution, with a median age of diagnosis at 74 years (range 17–100 years). The 5-year overall survival for the cohort was 66.3%, with a mean of 52.9% (95%CI = 5.14–5.44).

**Figure 1:**
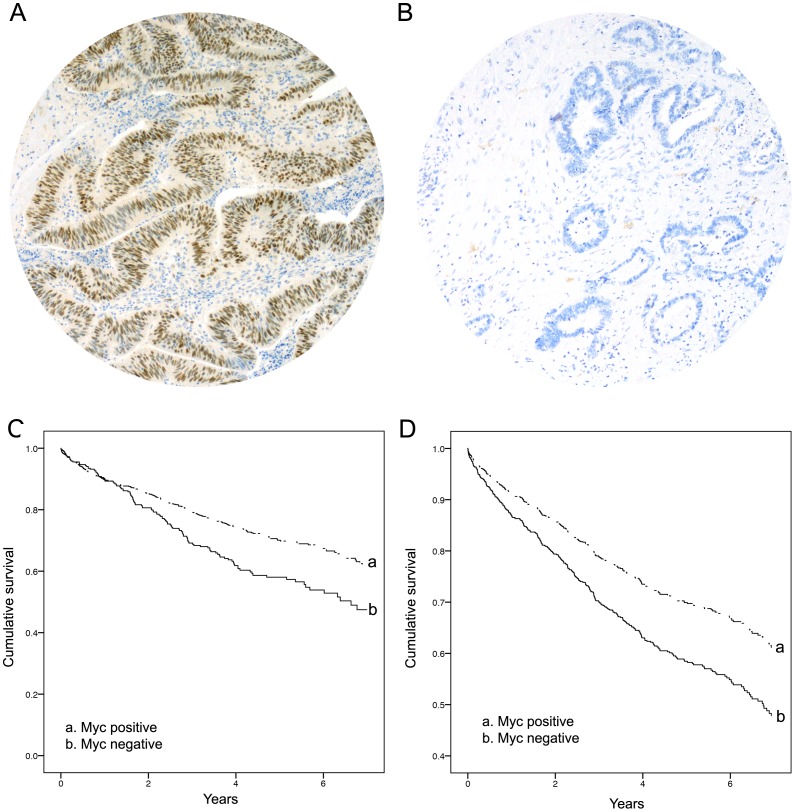
Panel A: Myc positive IHC staining; Panel B: Myc negative IHC staining; Panel C: Kaplan Meier analysis (Log Rank test p = 0.01); Panel D: Univariable Cox regression.

Myc IHC showed consistent nuclear immuno-localisation, and there was a visible distinction between cases which were negative (scored as negative for over-expression), and those which showed weak to strong staining in 10% or more neoplastic cells (scored as positive for over-expression). Examples of myc IHC staining characteristics are presented in Figure 1. The prevalence of MYC over-expression in CRC was 69%.

The clinical and pathological features of myc IHC negative (n = 441, 31.0%) and myc IHC positive (MYC over-expressed, n = 980, 69.0%) CRCs are summarized in Table 1. MYC over-expression was significantly associated with tumor stage and MMR/BRAF IHC phenotype, but not gender, anatomic location, histologic grade, presence or absence of lymphovascular space invasion, nor peritumoral lymphocyte reaction status. The relationship between MYC over-expression and age at diagnosis tended to significance (p = 0.07).

Table 2 shows the adjusted effect of of MMR/BRAF IHC phenotype on myc IHC status. MYC over-expression was strongly associated with the MMRd/BRAFV600E IHC phenotype [odds ratio = 2.17 (95%CI = 1.45–3.24), p<0.01].

The 5-year survival for CRCs with MYC over-expression was 93.2% (overall 50^th^ centile survival 3.06 years, interquartile range 0.56–5.22), compared with myc negative CRC of 57.3% (overall 50^th^ centile survival 2.32 years, interquartile range 0.31–4.26).

The effect of myc status on CRC overall survival is displayed in Figure 1. CRCs with MYC over-expression showed significantly better survival compared to myc negative cases (Log Rank test p<0.01), with a crude effect (univariable model) hazard ratio of 0.67 [(95%CI = 0.54–0.83), p<0.01]. This crude effect on overall survival remained significant even when stratified by MMR/BRAF IHC phenotype [hazard ratio = 0.68 (96%CI = 0.54–0.84), p<0.01].

In the full multivariable model, the adjusted effect of MYC over-expression on overall survival became insignificant [hazard ratio = 0.91 (95%CI = 0.69–1.20), p = 0.52] due to the dominant effect of age at diagnosis, tumor stage and lymphovascular space invasion status and the MMR deficient/BRAFV600E mutant IHC phenotype on survival (Table 3). Table 4 displays the full model including interaction between myc IHC and MMR/BRAF status.

**Table 3:** Multivariable Cox regression proportional hazards analysis of 1421 CRCs.

Variable	Multivariable analysis
	*HR (95%CI), p*
**Gender**	
Female	1.00
Male	1.25 (0.94–1.65), 0.12
**Age at diagnosis**	1.04 (1.03–1.05), <0.01
**Anatomic location**	
Left colon	1.00
Right colon	1.10 (0.83–1.45), 0.52
**Histologic grade**	
Low	1.00
High	1.28 (0.90–1.83), 0.18
**Lymphovascular space invasion**	
Absent	1.00
Present	1.60 (1.14–2.30), <0.01
**Peritumoral lymphocyte reaction**	
Absent	1.00
Present	1.93 (0.75–4.97), 0.18
**Overall Stage AJCC/TNM 7^th^ edition**	
I	1.00
IIA	2.22 (1.20–4.11), 0.01
IIB	2.67 (1.21–5.90), 0.02
IIC	11.91 (4.68–30/29), <0.01
IIIA	0.96 (0.27–3.39), 0.95
IIIB	2.70 (1.46–4.99), <0.01
IIIC	5.78 (3.06–10.89), <0.01
IVA	11.0 (4.61–26.27), <0.01
IVB	15.34 (6.47–36.37), <0.01
**MMR/BRAF IHC phenotype**	
MMRp/BRAFwt	1.00
MMRd/BRAFwt	0.58 (0.29–1.16), 0.13
MMRd/BRAFV600E	0.60 (0.37–0.97), 0.04
MMRp/BRAFV600E	1.07 (0.67–1.70), 0.78
Myc IHC status	
Negative	1.00
Positive	0.91 (0.69–1.20), 0.49

**Table 4:** Multivariable Cox regression proportional hazards analysis of 1421 CRCs including myc IHC status and MMR/BRAF IHC phenotype interaction terms.

Variable	Multivariable analysis
	*HR (95%CI), p*
**Gender**	
Female	1.00
Male	1.24 (0.93–1.64), 0.14
**Age at diagnosis**	1.04 (1.03–1.05), <0.01
**Anatomic location**	
Left colon	1.00
Right colon	1.11 (0.84–1.48), 0.47
**Histologic grade**	
Low	1.00
High	1.29 (0.90–1.85), 0.17
**Lymphovascular space invasion**	
Absent	1.00
Present	1.60 (1.14–2.23), <0.01
**Peritumoral lymphocyte reaction**	
Absent	1.00
Present	1.98 (0.77–5.13), 0.16
**Overall Stage AJCC/TNM 7^th^ edition**	
I	1.00
IIA	2.22 (1.20–4.11), 0.01
IIB	2.68 (1.21–5.93), 0.02
IIC	12.14 (4.77–30.88), <0.01
IIIA	0.97 (0.27–3.41), 0.96
IIIB	2.70 (1.46–4.99), <0.01
IIIC	5.83 (3.09–11.00), <0.01
IVA	10.84 (4.53–25.94), <0.01
IVB	15.67 (6.59–37.22), <0.01
**MMR/BRAF IHC phenotype**	
MMRp/BRAFwt	1.00
MMRd/BRAFwt	0.29 (0.07–1.21), 0.09
MMRd/BRAFV600E	0.77 (0.36–1.67), 0.77
MMRp/BRAFV600E	0.91 (0.47–1.75), 0.77
Myc IHC status	
Negative	1.00
Positive	0.87 (0.64–1.20), 0.39
Interaction terms	
Myc *MMRp/BRAFwt	1.00
Myc *MMRd/BRAFwt	2.77 (0.55–13.88), 0.22
Myc *MMRd/BRAFV600E	0.70 (0.28–1.74), 0.44
Myc *MMRp/BRAFV600E	1.38 (0.58–3.32), 0.47

MMRd – DNA mismatch repair deficient; MMRp – DNA mismatch repair proficient; BRAFwt – BRAF wild type; BRAFV600E – BRAFV600E mutant.

**Figure 2:**
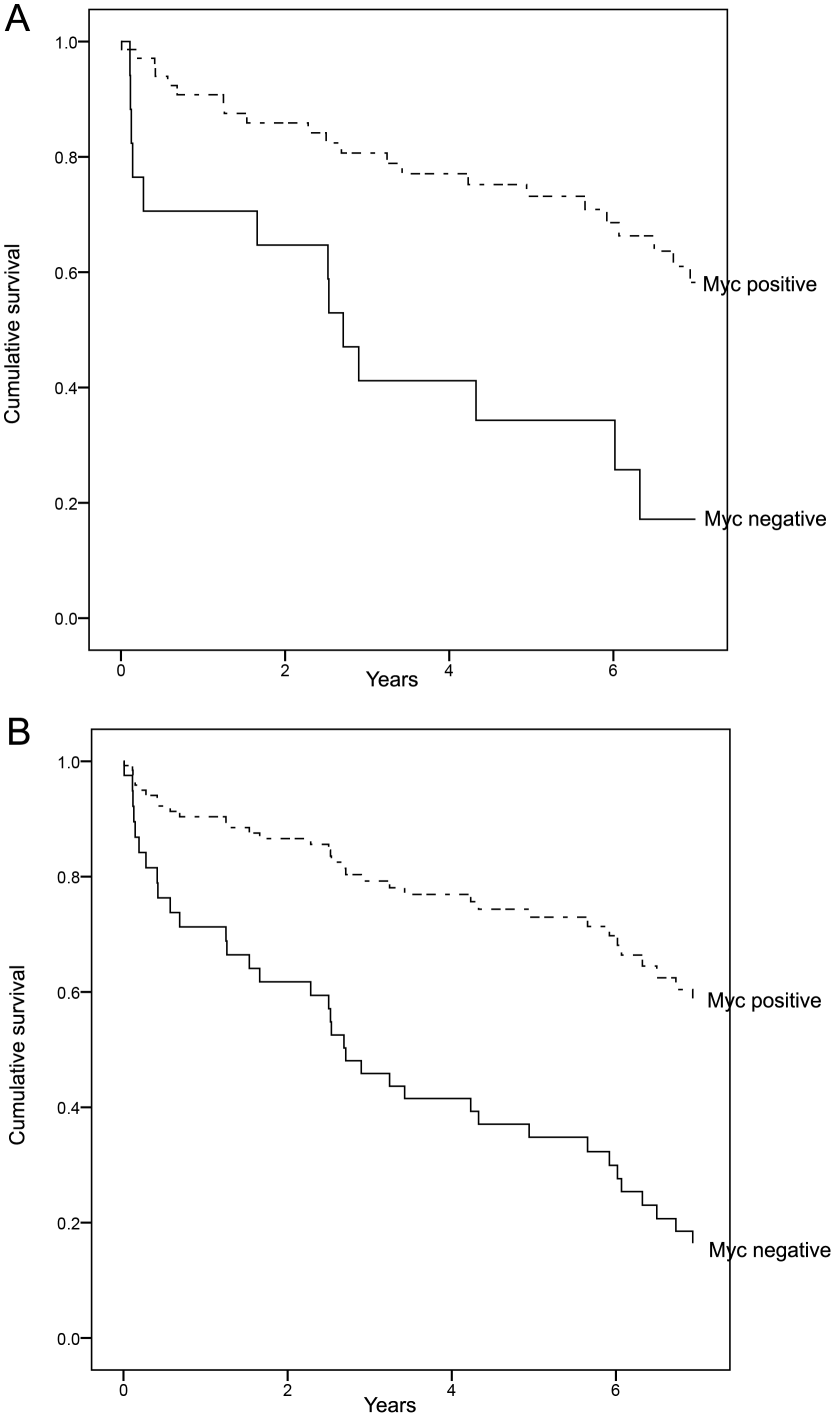
Myc IHC on whole section CRCs from 2004, confirming TMA findings. Panel A: Kaplan Meier curves showing superior overall survival of CRCs with MYC over-expression compared to myc negative CRCs (Log Rank test p<0.01); Panel B: Univariable Cox regression analysis showing MYC over-expression significantly correlated with improved overall survival [hazard ratio = 0.30 (95%CI = 0.15–0.60), p<0.01].

There was substantial agreement between TMA and whole section myc IHC scores (kappa = 0.742, p<0.01). All positive TMA cases were positive on whole sections. There were 11 discordant cases (negative on TMA, positive on whole section), all due to patchy staining. Univariable Cox regression was also performed on whole sections, confirming the improved overall survival of CRCs showing MYC over-expression (Figure 2).

## Discussion

Previous studies on MYC expression in CRC have been inconsistent and conflicting presumably due to the use of small cohorts (n ranging from 38 to 310) and the use of a variety of detection methods including IHC, fluorescent in-situ hybridization (FISH), RNA-based analysis and DNA copy number. [5], [13]–[17]. Older studies using IHC have been limited by the use of antibodies which demonstrated aberrant (cytoplasmic) immuno-localisation presumably due to poor specificity. [18].

The development and validation of a highly specific rabbit monoclonal antibody for myc has made a major impact in lymphoma diagnosis where the combination of MYC and BCL2 overexpression as determined by immunohistochemistry alone has been used to define a poor prognosis group of B-cell non Hodgkin lymphoma known as double hit lymphomas. [6], [7] Interestingly, in these lymphoma studies where myc IHC and FISH assessment have resulted in conflicting results, MYC expression as determined by IHC has been a better predictor of outcome than the previous gold standard FISH. Presumably this is because MYC over-expression can be due to a variety of cellular processes rather than just simple amplification.

In this context, our study, which is based on a very large and unselected cohort on 1421 CRC cohorts using this next generation of specific rabbit monoclonal antibody, demonstrates the high prevalence of MYC over-expression in CRCs, and more importantly, the potential role of myc as a powerful prognostic biomarker in CRCs. The fact that MYC over-expression is significantly associated with better overall survival in univariable analysis is perhaps surprising given current understanding of the tumorigenic potential of MYC dysregulation [1]–[4]. Only one previous study has shown this effect in CRC [5]. While there is insufficient data to postulate as to the underlying mechanism, the correlation between tumorigenic potential (oncogenesis) and survival, at least for MYC, is like to be more complex than initially thought.

In this study we have chosen to examine MYC over-expression in the context of CRCs grouped according to their MMR and BRAF status, rather than MMR and BRAF status individually. It is now well established that MMR and BRAF interact in complex ways in relation to their effect on survival. For example, in MMR proficient CRCs, the presence of BRAFV600E mutation confers a worse survival, whereas in MMR deficient CRCs, BRAFV600E is a marker of the methylator phenotype, a group of CRCs which show excellent prognosis [12], [19].

Interestingly, although MYC over-expression was strongly associated with the MMRd/BRAFV66E phenotype, their lack of interaction in the full multivariable model shows that MYC’s effect on survival cannot be explained entirely just by its association with MMR/BRAF status. This suggests that the predictive effect of MYC over-expression on survival is mediated in part, by mechanisms independent of MMR/BRAF status.

One of the major drawbacks of our study is the over-simplification by which MYC over-expression is determined. The semi-quantitative nature of IHC is such that changes in IHC intensity need to be significant (in the logarithmic scale, otherwise known as the Weber-Fechner law) in order that the human eye be able to appreciate a change. This method of IHC scoring does however lend itself to more straightforward correlative analysis such as binary logistic regression, and is the predominant methodology by which most IHC is scored in the diagnostic clinical setting. Significantly, despite this drawback we were able to demonstrate significant overall survival differences on univariable analysis of myc IHC status.

Another significant drawback is the lack of disease specific outcomes and cancer specific mortality in our study cohort. Future studies employing these endpoints will provide important information which may significantly inform on clinical practice.

In summary, our study shows that myc status, as determined by IHC using a simple semi-quantitative scoring methodology readily deployed in the clinical setting can be used as a predictor of prognosis in CRC. The refinement of this predictive utility awaits further elucidation in cohorts with disease specific outcomes.
